# Pulmonary Function, Mental and Physical Health in Recovered COVID-19 Patients Requiring Invasive Versus Non-invasive Oxygen Therapy: A Prospective Follow-Up Study Post-ICU Discharge

**DOI:** 10.7759/cureus.17756

**Published:** 2021-09-06

**Authors:** Amarjyoti Hazarika, Varun Mahajan, Kamal Kajal, Ananya Ray, Karan Singla, Inderpaul S Sehgal, Ashish Bhalla, Shubh M Singh, Naveen B Naik, Narender Kaloria, Kulbhushan Saini, Ajay Singh, Ganesh Kumar, Indranil Biswas, Shiv L Soni, Hemant Bhagat, Yadvender Singh, Goverdhan D Puri

**Affiliations:** 1 Anesthesia and Intensive Care, Postgraduate Institute of Medical Education and Research, Chandigarh, IND; 2 Anesthesia and Intensive care, Postgraduate Institute of Medical Education and Research, Chandigarh, IND; 3 Pulmonary Medicine, Postgraduate Institute of Medical Education and Research, Chandigarh, IND; 4 Internal Medicine, Postgraduate Institute of Medical Education and Research, Chandigarh, IND; 5 Psychiatry, Postgraduate Institute of Medical Education and Research, Chandigarh, IND; 6 Anaesthesia and Intensive Care, Postgraduate Institute of Medical Education and Research, Chandigarh, IND; 7 Hospital Adminstration, Postgraduate Institute of Medical Education and Research, Chandigarh, IND

**Keywords:** covid-19, acute respiratory distress syndrome [ards], follow-up study, respiratory function tests, health-related quality of life, 6-minute walk test

## Abstract

Background

Survivors of COVID-19 pneumonia may have residual lung injury and poor physical and mental health even after discharge. We hypothesized that COVID-19 severe acute respiratory distress syndrome (ARDS) patients needing mechanical ventilation may be at a greater risk of deterioration in pulmonary function, mental health, and quality of life (QOL). This study analyses the differences in pulmonary function, mental health, and QOL after recovery, in patients having received non-invasive oxygen therapy versus invasive mechanical ventilation during ICU stay.

Methods

Patients aged >18 years, who had completed 3 months post ICU discharge, with moderate to severe COVID-19 ARDS, were consecutively enrolled from May 1 to July 31, 2021. Patients were allocated into Group A - having required high flow nasal cannula (HFNC)/non-invasive ventilation (NIV) and Group B - having received invasive mechanical ventilation. Pulmonary function tests, 6-minute walk test (6-MWT), and health-related quality of life were compared.

Results

Of the 145 eligible patients, 31 were lost to follow-up and 21 died. Seventy-four patients were allocated into Groups A (57 patients) and B (17 patients). In Group A, abnormal forced expiratory volume in first second (FEV_1_), forced vital capacity (FVC), forced expiratory flow in mid-half of FVC (FEF_25-75_), and peak expiratory flow rate (PEFR) values were obtained in 27 (47.37%), 43 (75.44%), 11 (19.3%), and 25 (43.86%) patients, and in Group B, in 13 (76.47%), 17 (100%), 1 (5.88%), and 8 (47%) patients, respectively. No patient had abnormal FEV_1_/FVC. All Group B patients had a restrictive pattern in spirometry as compared to 77% in Group A. Group B had a lower arterial partial pressure of oxygen (PaO_2_) (p=0.0019), % predicted FVC (p<0.0001), % predicted FEV_1_ (p=0.001), and 6-MWT distance (p<0.001). The physical component score in the short-form survey 12 questionnaire was higher in group A, p<0.001, whereas the mental component score was comparable.

Conclusions

Patients requiring invasive mechanical ventilation (MV) have a greater risk of impaired pulmonary function and reduced QOL post-ICU discharge. This warrants a greater need for following these patients for better rehabilitation.

## Introduction

The coronavirus disease 2019 (COVID-19) pandemic has presented a challenge to clinicians all over the world. Severe acute respiratory syndrome coronavirus 2 (SARS-CoV-2), causing COVID-19 disease, primarily affects the lungs. The clinical manifestations range from asymptomatic carriage to atypical pneumonia and acute respiratory distress syndrome (ARDS) [1]. Most patients requiring intensive care unit (ICU) admission, require invasive or non-invasive ventilatory support to maintain oxygenation. To reduce this work of breathing associated with hypoxemia, non-invasive oxygen therapy in the form of high flow nasal cannula (HFNC) and non-invasive ventilation (NIV) are administered. NIV may improve the long-term outcome in carefully selected patients [2]. In certain cases, the use of HFNC and NIV may circumvent the need for intubation, reducing the hazards associated with intubation and invasive mechanical ventilation (MV), such as ventilator-associated pneumonia, lung injury, acute kidney injury, hemodynamic instability, etc. This is especially important in COVID-19 treatment since patients are administered immunosuppressive therapy (e.g., steroid) as part of the standard treatment regime [3]. Nonetheless, with the use of NIV for COVID-19 ARDS, a failure rate of 40-50% may be expected [4]. The patients who fail an NIV trial frequently require mechanical ventilation (MV). Literature suggests that the rate of MV among COVID patients ranges from 29.1% to 89.9% [5].

After ICU discharge, a significant percentage of patients have been found to suffer from muscle fatigue as well as physical, mental, and cognitive complications [6]. Fifty percent of all patients, irrespective of age, who require MV develop post-intensive care syndrome [7]. Cognitive impairment and quality of life also remain impaired amongst the survivors. Prolonged mechanical ventilation has been shown to deteriorate the health-related quality of life (HRQOL) and physical function in survivors [8]. Screening of COVID-19 patients post-discharge has also demonstrated signs of anxiety and depression [9]. Addressing these concerns now constitutes a new challenge in the aftermath of the pandemic.

In this study, we have described the extent of residual abnormalities in lung function, and the physical and psychological stress at 3 months post-discharge from the ICU in a prospective cohort of patients who had received either NIV or MV during their ICU stay.

## Materials and methods

This single-center, non-randomized, prospective cohort study was carried out at a tertiary care center from May 1 to July 31, 2021. The study was approved by the Institutional Ethics Committee (INT/IEC/2021/SPL-340) and registered in the Clinical Trial Registry of India (www.ctri.nic.in) before commencement (CTRI/2021/04/032768). Written informed consent was obtained from each participant.

Inclusion criteria 

1. All consecutive COVID-19 positive, diagnosed by reverse transcription polymerase chain reaction (RT-PCR) test patients, aged 18 years and above.

2. Partial pressure of arterial oxygen concentration to fraction of inspired oxygen, PaO_2_:FiO_2_ ≤200 mmHg at admission to ICU.

3. Completed 3 months post ICU discharge.

Exclusion criteria 

1. In-hospital mortality or death within 3 months of hospital discharge. 

Outcomes measures

Primary Outcome

To compare FVC (forced vital capacity), forced expiratory volume in the first second (FEV_1_), forced expiratory flow in mid-half of FVC (FEF_25-75_), and peak expiratory flow rate (PEFR) and FEV_1_/FVC ratio at 3 months post ICU discharge in patients who received non-invasive oxygen therapy versus invasive mechanical ventilation during their ICU stay. 

Secondary Outcomes

Comparison of six-minute walk test (6-MWT), health-related quality of life (HRQOL), medications received at discharge, and readmission post-discharge (within the 3 month period) between the two groups.

Allocation

Based on the mode of oxygenation received in the ICU, the patients were divided into two groups:

Group A: COVID-19 positive patients having received non-invasive oxygen therapy via high-flow nasal cannula (HFNC) or non-invasive ventilation (NIV). 

Group B: Patients with COVID-19 ARDS who received invasive mechanical ventilation (MV).

Study protocol

Patients were enrolled as per the inclusion criteria from the ICU registry and were contacted telephonically and were requested to attend the follow-up clinic, upon completion of the 3 months period post-ICU discharge. 

Respiratory Parameters

Spirometry and room air arterial blood gas (ABG) analysis were performed for each patient. In spirometry, the percentage predicted values for FVC, FEV1, FEV_1_/FVC ratio, FEF_25-75,_ and PEFR were measured. Spirometry was performed in accordance with the American Thoracic Society and European Respiratory Society (ATS/ERS) task force guidelines [10]. Spirometry was performed seven times in each patient and the average of three best performances were recorded. The technician and physician guiding the patients wore an N-95 respirator mask, face shield, and a disposable gown. The Easy on-PC portable spirometer (TrueFlow™ technology, NDD Medizinitechnik, Zurich Switzerland) was used to perform spirometry. FVC, FEV_1_, PEFR, FEF_25-75,_ and the ratio of FEV_1_/FVC were recorded.

Six-Min Walk Test (6-MWT)

The 6-MWT reflects the effort tolerance of the patient. The 6-MWT was performed in accordance with the ATS statement [11] on a level surface and was supervised by a physical therapist who was blinded to patient allocation. The test was performed on room air and a drop in oxygen saturation to less than 94% or a fall > 3% from the baseline at the end of the test was considered a positive test [12]. Oxygen saturation (SpO_2_) was measured by pulse oximeter at the time of commencement and at the completion of the 6-MWT. The arterial partial pressure of oxygen (PaO_2_) and arterial partial pressure of carbon dioxide (PaCO_2_) were noted from the ABG prior to commencing the 6-MWT. The total distance covered was also measured.

Health-Related Quality of Life (HRQOL)

HRQOL measures an individual's subjective physical and mental well-being over time following recovery from a debilitating illness. To measure HR-QOL, the SF-12 questionnaire, which deals in physiological, psycho-social, and environmental factors related to a patient’s health status, was used. These questions were summarised into two scales: a physical component score (PCS) and a mental component score (MCS) [13]. Both the scores range from 0 to 100, and a higher score indicates better health. Since there is no reference score for India, a mean score of 50 was taken from across 10 countries [14].

Statistical analysis

The normality of the data was assessed by the Shapiro-Wilk test. Parametric data has been expressed as mean ± SD and analysed using the Student's t-test. Non-parametric data has been expressed as median inter-quartile range (IQR) and analysed using the Mann-Whitney U test. Categorical variables have been expressed as absolute number (percentage) and analysed with Pearson Chi-square or Fisher’s exact test as appropriate. All the statistical analysis has been done using the software IBM® SPSS® version 24 (IBM Corp, Armonk, USA).

## Results

**Figure 1:**
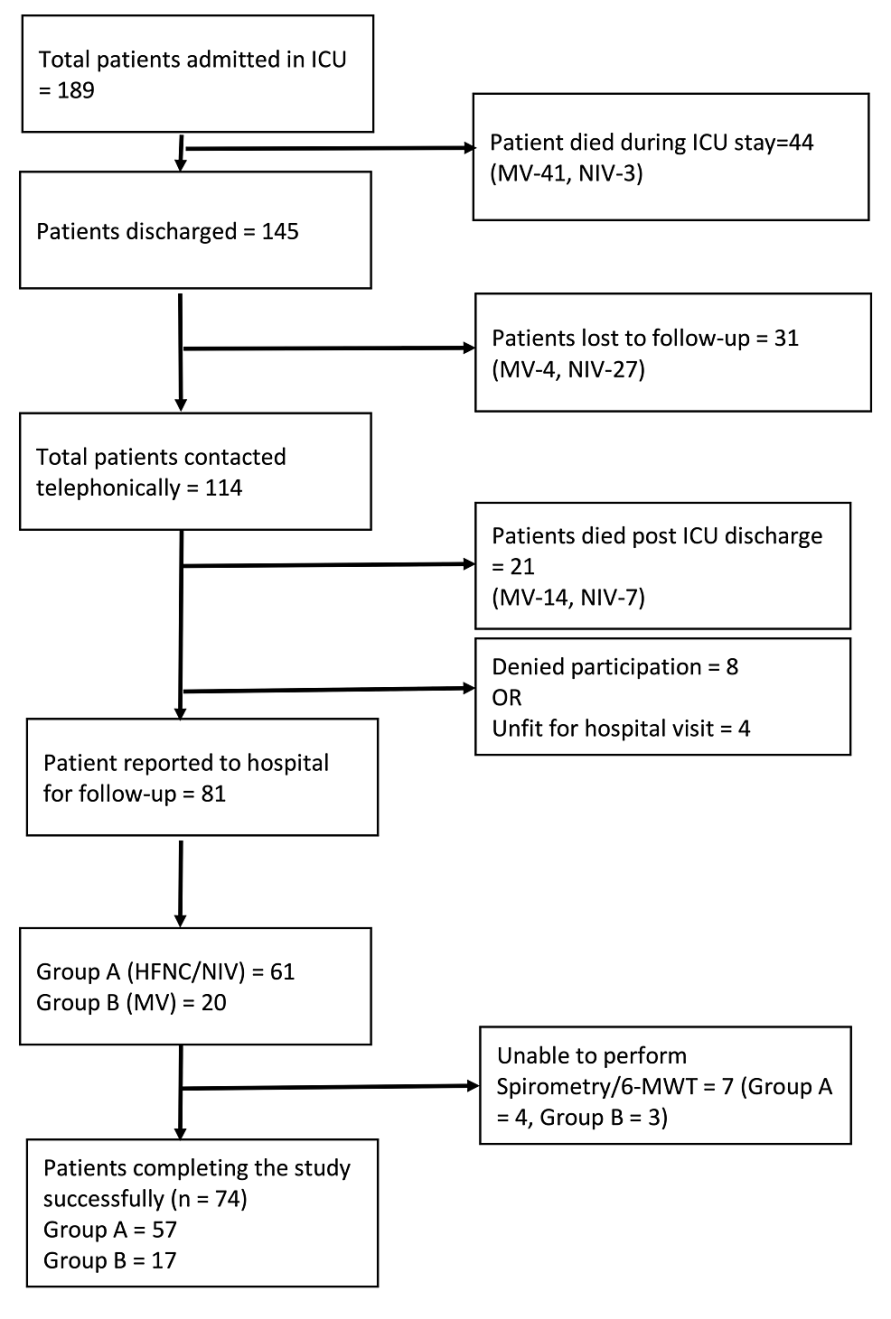
Flow diagram of the study. *Abbreviations:* ICU – 6-MWT – 6-minute walk test Intensive care unit, HFNC – High flow nasal cannula, MV - Invasive mechanical ventilation and NIV – Non-invasive ventilation.

Of the 189 patients admitted to ICU from January 1 to April 30, 2021, 145 were discharged. Of them, 31 were lost to follow-up. The remaining 114 patients were contacted telephonically at 3 months post-discharge i.e., from May 1 to July 31, 2021. Twenty-one patients had died within the first 3 months post ICU discharge. Among the 93 survivors, 81 reported to the hospital to participate in the study. Of them, only 74 patients could successfully complete the study (Figure 1).

The demographic data in both groups were comparable except for age. None of the participants had pulmonary disease. The mean ± SD age of patients in Group A was 53.18 ± 13.63 years and for Group B was 44.24 ± 11.6 years, respectively, p=0.008 (Table 1). The (mean ± SD) P/F ratio at admission in Group A was 116.51 ± 38.24 mmHg and in Group B was 83 ± 23.5 mmHg, p=0.001. The Sequential Organ Failure Assessment (SOFA) score at admission was 3 (2,3.5) for Group A and 4 (3,4) for group B (p <0.0001) (Table 1, 2 ).

**Table 1:** Demographic and baseline characteristics of patients. Parametric variables analysed using Student's t-test and expressed as mean ± standard deviation (SD). Non-parametric variables analysed using Mann-Whitney U test and expressed as median and inter-quartile range (IQR). Nominal variables analysed using Pearson’s Chi-squared or Fisher’s exact test and expressed as absolute number and percentage. P-value<0.05 is significant. *Abbreviations:* BMI – Body mass index, PaO_2_:FiO_2_ – Ratio of partial pressure of oxygen to fraction inspired of oxygen, SOFA – Sequential organ function assessment score.

	Overall (n=74)	Non-invasive therapy (Group A) (n=57)	Invasive mechanical ventilation (Group B) (n=17)	p-value
Age	50 ± 13.78	53.18 ± 13.63	44.24 ± 11.6	0.008
Sex-Males (%)	48 (64.9%)	37 (64.9%)	11 (64.7%)	0.988
Height	169.11 ± 5.29	169.44 ± 5.4	168 ± 4.92	0.329
Weight	74.3 ± 10.5	75.46 ± 10.14	70.41 ± 11.07	0.082
BMI (kg/m^2^)	25.96 ± 3.39	26.28 ± 11.31	24.89 ± 3.34	0.140
PaO_2_:FiO_2_ at admission (mmHg)	108.81 ± 38	116.51±38.24	83 ± 23.5	0.001
SOFA score at admission	3 (3,4)	3 (2,3.5)	4 (3,4)	<0.0001
Comorbidities				
Hypertension	30 (40.54%)	24 (42.1%)	6 (35.29%)	0.417
Diabetes	30 (40.54%)	23 (40.35%)	7 (41.18%)	0.583
Hypothyroidism	4 (5.4%)	3 (5.26%)	1 (5.88)	0.657
Obesity (BMI>30 kg/m^2^)	7 (9.46%)	6 (10.53%)	1 (0.6%)	0.490
Chronic kidney disease	2 (2.7%)	2 (3.51%)	0	0.591

**Table 2:** Showing the spirometry values, blood gases, 6-MWT and Short Form health survey-12 (SF-12) scores. Spirometry values are presented as percentage of predicted values. Minimum PaO_2_:FiO_2_ ratio in Group A was the least PaO_2_:FiO_2_ ratio attained by the patient during ICU stay. Minimum PaO_2_:FiO_2_ ratio in Group B is the PaO_2_:FiO_2_ ratio at the time of intubation. Abbreviations: 6-MWT – 6-Minute walk test, BMI - Body mass index, F – Female, FEV_1 _– Forced expiratory volume in first second of FVC, FVC – Forced vital capacity, FEF_25-75_ – Forced expiratory flow in mid-half of FVC, M – Male, MCS – Mental component score, PaCO_2_ – Arterial partial pressure of carbon dioxide in mmHg, PaO_2_ – Arterial partial pressure of oxygen in mmHg, PEFR – Peak expiratory flow rate, PaO_2_:FiO_2_ -  Ratio of arterial partial pressure of oxygen to fractional of inspired oxygen, PCS – Physical component score, SOFA – Sequential organ function assessment score.

S. no.	Group	Age in years	Sex	Height in cm	Weight in Kgs	BMI (Kg/m^2^)	ICU stay	PaO_2_:FiO_2_ at admission (mmHg)	SOFA score at admission	Minimum PaO_2_:FiO_2_	FVC%	FEV_1_%	PEFR%	FEF_25-75_%	FEV_1_/FVC %	Interpretation	PaO_2 _(mmHg)	PaCO_2 _(mmHg)	6-MWT	% Drop In SpO_2_(%)	MCS	PCS
1	A	68	M	172	100	33.80	8	55	2	55	76	89	132.1	124.3	117	Restrictive	90.2	35.4	294	1	60.75	56.5
2	A	35	F	167	98	35.14	6	131	2	108	48	54	91.4	64.1	112	Restrictive	92.6	32.7	182	3	57.38	51.41
3	A	67	F	160	71	27.73	11	100	3	91	57	72	83.2	116.3	126	Restrictive	86.1	30.9	238	3	60.75	56.7
4	A	69	M	177	77	24.58	5	102	3	85	92	115	74.6	143.8	126	Normal	89.8	38	308	1	59.25	52.76
5	A	34	M	174	89	29.40	6	126	3	88	59	63	104.4	104.2	106	Restrictive	77.2	32.4	294	2	46.7	43.35
6	A	34	M	167	70	25.10	8	133	5	104	59	58	72.2	100.6	98.5	Restrictive	88.3	43.7	322	1	54.25	51.58
7	A	35	M	175	78	25.47	6	64	4	62	70	58	78.4	91.2	82	Restrictive	74.2	32.3	285	1	55.39	47.4
8	A	49	M	168	80	28.34	8	80	4	80	82.6	59	56.5	59.7	72	Obstructive	67.7	36.5	281	1	60.2	41.77
9	A	32	M	156	65	26.71	5	54	2	54	61.3	70	72.1	85.2	110	Restrictive	75.1	33.2	315	1	57.82	55.5
10	A	37	M	169	75	26.26	6	82	4	82	64.66	69	97.6	93.4	106	Restrictive	74.2	36.7	298	2	58.52	52.4
11	A	67	F	161	70	27.01	14	106	3	100	71	73	87.4	97.1	102	Restrictive	66.4	37.1	192	3	54.62	40.49
12	A	53	F	170	110	38.06	6	142	2	135	74	91	95.3	101.2	123	Restrictive	81.7	34.9	317	1	57.82	55.5
13	A	74	F	167	54	19.36	10	92	4	92	91.6	89	93.6	95.5	97.6	Normal	72.6	34	289	1	54.71	41.91
14	A	50	M	160	80	31.25	6	90	2	84	94.93	83.5	78.1	97.5	103	Normal	65.6	38	234	2	51.82	38.33
15	A	73	M	172	72	24.34	8	175	3	120	61.3	79.04	54.98	64.6	122	Restrictive	73.8	35.2	256	1	64.5	32.94
16	A	55	M	165	55	20.20	11	97	4	97	60.8	74.5	84.2	110.2	122	Restrictive	70.3	36.6	250	1	61.73	40.2
17	A	70	M	162	85	32.39	12	62	4	62	63	81	83.4	98.3	128	Restrictive	66.1	37.5	246	2	62.27	39.58
18	A	56	M	172	77	26.03	5	116	3	93	78	91.16	79.1	77.9	117	Restrictive	79.8	36.2	261	1	51.48	49.42
19	A	52	M	167	79	28.33	6	92	4	80	76.5	87	82.2	83.5	114.1	Restrictive	71.1	34.4	266	1	60.92	47.24
20	A	47	M	174	81	26.75	9	153	3	98	74.2	82.5	88.1	87.2	111.2	Restrictive	87.4	32.2	285	1	47.31	55.16
21	A	25	F	169	64	22.41	6	189	3	105	79.3	79.3	96	72.6	99.8	Restrictive	79.2	38.5	274	1	51.99	55.3
22	A	49	M	175	78	25.47	5	182	2	146	65.2	66.8	95.4	91.5	103	Restrictive	75.2	35.5	262	1	38.22	57.56
23	A	64	M	169	68	23.81	6	101	3	101	71.5	79.3	91.1	84.6	111.5	Restrictive	82.1	36.9	287	1	57.09	54.77
24	A	59	M	170	67	23.18	7	144	3	87	78.8	91.33	87.2	78.2	116.4	Restrictive	74.6	32.5	248	1	56.71	48.25
25	A	55	M	175	66	21.55	14	117	2	74	61.9	65.1	86.1	89.6	105.7	Restrictive	75.7	38.8	269	2	52.49	57.12
26	A	46	M	174	77	25.43	12	100	3	78	74.2	86.6	88.6	86.2	116.8	Restrictive	69.2	33.2	254	2	55.04	45.1
27	A	44	F	166	75	27.22	9	108	3	94	77.1	89.6	97.2	91.4	116.3	Restrictive	86.3	32.1	315	1	55.67	56.2
28	A	62	F	165	72	26.45	10	83	2	80	70.2	78.9	92	97.4	112.6	Restrictive	89.2	34.8	271	1	57.29	53.17
29	A	67	F	169	82	28.71	8	69	2	65	69.8	76.92	78	78.9	110.3	Restrictive	88.6	35.1	277	1	57.39	51.47
30	A	32	M	172	88	29.75	9	184	3	160	64.9	66.74	58.3	88.8	103.1	Restrictive	85.7	36.9	281	1	57.09	54.77
31	A	63	M	170	78	26.99	12	150	2	116	77.5	78.8	49.3	78.6	102.2	Restrictive	91.2	37.8	309	1	59.77	54.83
32	A	67	M	176	82	26.47	8	73	4	72	79.4	99	68.9	71.5	126.4	Restrictive	88.5	33.3	294	1	59.04	54.11
33	A	74	M	171	75	25.65	10	110	3	84	80.3	105.3	78.6	98.6	130.7	Normal	82.6	35.7	285	1	57.49	51.93
34	A	71	F	165	68	24.98	13	127	3	79	64.6	70.2	82.3	89.6	108.6	Restrictive	78.1	34.3	254	1	59.25	52.76
35	A	62	M	169	72	25.21	11	64	2	64	62.7	66.5	84.6	95.2	106.6	Restrictive	92.5	36	318	1	59.77	54.83
36	A	53	F	164	65	24.17	8	154	3	86	79.4	95	78.2	106.2	119.5	Restrictive	87.8	37.2	295	1	60.92	47.24
37	A	58	F	167	62	22.23	9	100	3	98	81.5	98.3	91.5	100.7	120.9	Normal	78.2	34.5	274	1	62.27	39.58
38	A	60	M	172	77	26.03	16	92	4	77	77.6	95.73	77.6	97.5	123.9	Restrictive	77.5	40.6	263	1	61.02	45.54
39	A	46	M	177	76	24.26	14	170	2	118	85.6	102.4	69.5	99.2	119.8	Normal	81.2	38.6	287	1	57.09	54.77
40	A	73	F	160	68	26.56	9	190	3	148	66.2	65.2	81.9	74.6	98.7	Restrictive	85.6	38.1	301	1	56.37	52.74
41	A	35	M	176	84	27.12	7	132	2	94	84.2	97.4	84.9	87.2	115.7	Normal	95.8	36.4	324	1	58.74	55.92
42	A	46	M	174	78	25.76	5	115	3	101	86.1	103.9	74.6	98.2	120.5	Normal	90.5	35.6	317	1	60.75	56.57
43	A	53	M	169	80	28.01	9	83	4	82	77.8	84.02	75.9	94.3	108.5	Restrictive	95.2	37.2	324	1	57.88	56.81
44	A	67	M	173	75	25.06	12	172	2	94	71.8	71.3	58.6	89.6	99.5	Restrictive	84.9	41.4	288	1	56.56	52.45
45	A	65	F	165	68	24.98	13	110	3	85	80.2	88.8	66.3	92.4	110.7	Normal	71.5	32	266	1	53.46	47.86
46	A	58	F	166	68	24.68	10	165	3	92	68.6	72.45	78.2	78.6	105.8	Restrictive	84.1	38.6	283	1	59.96	47.42
47	A	41	F	163	80	30.11	14	89	4	89	71.5	83	84.3	94.2	116.3	Restrictive	88.5	37.1	308	1	55.04	53.77
48	A	43	M	170	85	29.41	8	136	3	112	79.3	92.1	89.2	115.2	116.1	Restrictive	92.6	38.6	337	1	60.75	56.57
49	A	33	F	164	65	24.17	19	118	3	79	84.6	95.7	84.1	108.7	112.9	Normal	79	32.4	273	1	47.8	56.77
50	A	28	M	175	82	26.78	9	127	2	96	79.5	92.5	79.5	103.9	115.8	Restrictive	92.4	38.6	322	1	47.04	58.91
51	A	35	M	177	79	25.22	8	105	3	83	81.2	92.1	81.3	100.6	113.6	Normal	88.7	37.1	292	1	59.77	54.83
52	A	61	F	166	68	24.68	15	59	4	57	75.4	78.5	77.4	99.5	104.1	Restrictive	83.5	36.3	304	1	57.42	55.5
53	A	52	F	167	65	23.31	11	64	4	64	70	75.5	75.3	101.9	107.5	Restrictive	78.3	38.1	312	1	57.82	55.5
54	A	56	F	166	60	21.77	10	183	3	124	82.4	90	80.2	98.6	109.2	Normal	74.8	33.6	304	1	57.19	53.07
55	A	49	M	178	75	23.67	10	138	3	114	78.4	88.1	91.2	95.2	112.5	Restrictive	70.8	36.4	295	1	56.56	52.45
56	A	50	M	180	83	25.62	9	168	2	137	68.6	75.4	89.5	107.6	110.3	Restrictive	95.7	34.3	336	1	60.75	56.57
57	A	72	M	179	80	24.97	11	118	2	96	82.3	93.8	79.4	101.3	113.6	Normal	81.5	38.2	311	1	57.92	53.79
58	B	40	M	175	98	32.00	27	115	3	61	69	83	103	149	121.26	Restrictive	86	34	280	1	61.12	49.3
59	B	23	F	160	65	25.39	22	78	4	72	75	83	67.19	98	111.38	Restrictive	62	38	196	1	60.66	39.7
60	B	36	M	164	77	28.63	28	63	4	62	66	72	76	92	109.7	Restrictive	84	38	182	1	61.39	40.42
61	B	35	M	165	57	20.94	16	57	4	57	58	65	30	55	109.34	Restrictive	93	40	154	2	57.37	44.61
62	B	30	F	164	49	18.22	11	102	3	77	73.35	77	91	109	102.99	Restrictive	77	36	210	1	55.19	45.34
63	B	48	M	170	61	21.11	19	116	3	58	52.9	59.19	75.96	109	120.43	Restrictive	67	35	126	2	46.57	35.2
64	B	26	F	166	63	22.86	17	80	4	75	49.8	61.1	82.3	96	93.33	Restrictive	78	33	182	1	51.53	28.22
65	B	54	M	174	75	24.77	23	62	4	62	62.8	67.67	87.2	105	107.77	Restrictive	81	36	210	1	56.12	48.12
66	B	47	M	170	82	28.37	24	54	4	54	59.4	65.86	79.4	97	111.16	Restrictive	75	34.5	224	1	62.48	42.43
67	B	63	M	172	78	26.37	18	79	4	68	77.3	87.45	81.6	99	113.55	Restrictive	79	37	196	1	58.18	44.55
68	B	55	M	178	69	21.78	21	98	4	78	56.2	61.44	77.9	103	109.68	Restrictive	71	34	168	2	55.95	33.99
69	B	58	M	168	76	26.93	25	100	3	77	61.8	66.77	85.3	96	108.41	Restrictive	69	39	168	2	49.04	41.18
70	B	52	F	165	72	26.45	16	125	3	71	67.5	75.8	78.1	101	112.48	Restrictive	74	32	154	3	58.11	35.83
71	B	44	M	162	68	25.91	28	60	4	58	74.9	80.0	74.6	107	106.69	Restrictive	76	36	210	1	59.23	37.91
72	B	39	F	164	62	23.05	32	90	4	78	63.7	68.61	91.5	96.5	107.65	Restrictive	82	34	196	1	46.89	41.88
73	B	34	F	168	70	24.80	9	50	4	50	48.3	51.83	88.6	98.1	107.66	Restrictive	71	38	154	2	53.28	39.55
74	B	51	M	171	75	25.65	15	82	4	64	57.4	66.05	80.5	106	115.29	Restrictive	68	41	126	3	46.5	37.9

Primary outcome: respiratory parameters 

**Figure 2:**
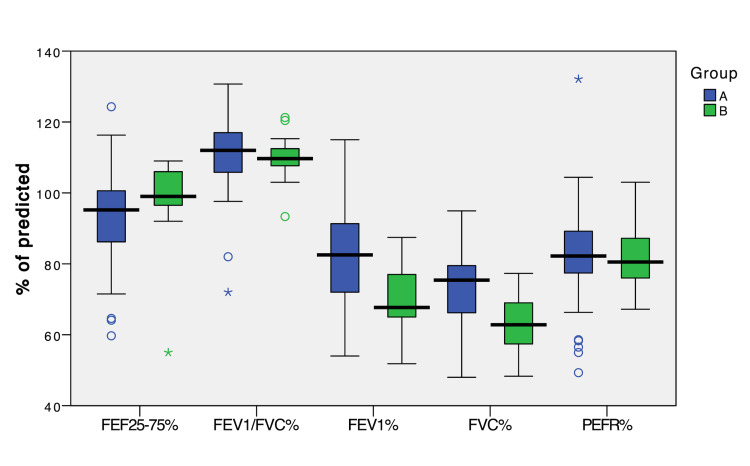
Clustered box plot showing percentage of predicted values for spirometry results. *Abbreviations:* 6-MWT – FEV_1_ – Forced expiratory volume in first second of FVC, FVC – Forced vital capacity, FEF_25-75_ – Forced expiratory flow in mid-half of FVC and PEFR – Peak expiratory flow rate.

**Figure 3:**
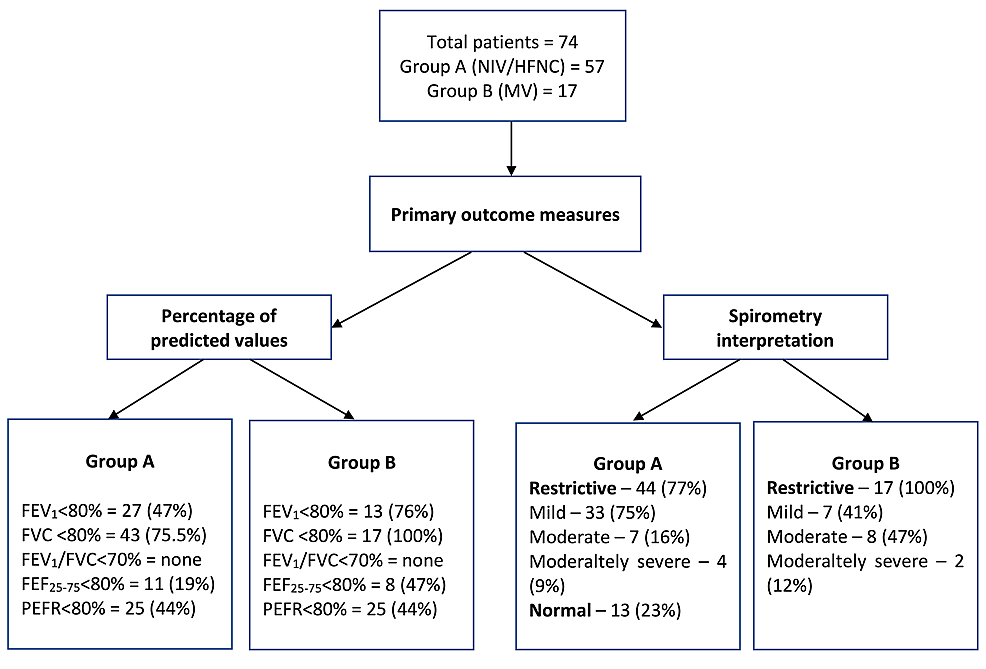
Summary of primary outcome measures. *Abbreviations:* HFNC – High flow nasal cannula, FEV_1_ – Forced expiratory volume in first second of FVC, FVC – Forced vital capacity, FEF_25-75_ – Forced expiratory flow in mid-half of FVC, MV – Invasive mechanical ventilation, NIV – Non-invasive ventilation and PEFR – Peak expiratory flow rate.

Of the 57 patients in Group A, the abnormal FEV_1_, FVC, FEF_25-75_ and PEFR values were obtained in 27 (47.37%), 43 (75.44%), 11 (19.3%) and 25 (43.86%) patients, respectively. None of the patients had abnormal FEV_1_/FVC values. On the interpretation of spirometry parameters, 44 (77.19%) had restrictive pattern while 13 (22.8%) patients had normal pulmonary function. In those who had a restrictive pattern, 30 patients received both HFNC and NIV whereas 14 patients received only HFNC during ICU stay. In terms of the severity of restriction, 33 (75%) patients had mild restriction, 7 (16%) moderate and 4 (9%) moderately severe restriction on spirometry (Table 3 and Figure 2, 3).

**Table 3:** Primary and secondary outcomes of the study. Parametric variables analysed using Students t-test and expressed as mean ± standard deviation (SD). Non-parametric variables analysed using Mann-Whitney U test and expressed as median and inter-quartile range (IQR). Nominal variables analysed using Pearson’s Chi squared or Fisher’s exact test and expressed as absolute number and percentage. P value<0.05 is significant. *Abbreviations:* 6MWTD – 6-Minute walk test distance, ICU – Intensive care unit, FEV_1_ – Forced expiratory volume in first second of FVC, FVC – Forced vital capacity, FEF_25-75_ – Forced expiratory flow in mid-half of FVC, MCS – Mental component score, PaCO_2_ – Arterial partial pressure of carbon dioxide, PaO_2_ – Arterial partial pressure of oxygen, PEFR – Peak expiratory flow rate, PCS – Physical component score.

Parameters	Overall (n=74)	Non-invasive therapy (Group A) (N-57)	Invasive ventilation (Group B) (N- 17)	p-value
FEV_1_ (L)	2.35 (1.92,2.76)	2.43 (1.93,2.93)	2.16 (1.88,2.52)	0.11
FEV_1_ (% of predicted)	78.97 (67.46,89.7)	82.5 (71.65,91.72)	67.67 (63.22,78.5)	0.001
FVC (L)	2.65 (2.16,3.13)	2.8 (2.21,3.2)	2.39 (2.05,2.78)	0.049
FVC (% of predicted)	71.65 (62.95,79.3)	75.4 (65.7,79.85)	62.8 (58.8,71.18)	<0.0001
PEFR (L/sec)	6.21 (5.26,7.18)	6.13 (5.15,7.42)	6.55 (5.75,7.09)	0.572
PEFR (% of predicted)	81.75 (75.99,88.75)	82.2 (76.65,89.35)	80.5 (75.98,87.9)	0.639
FEF_25-75_	2.76 (2.19,3.47)	2.67 (2.13,3.5)	3.33 (2.67,3.49)	0.055
FEF_25-75 _(% of predicted)	97 (88.4,101.45)	95.2(85.7,100.65)	99 (96.25,106.5)	0.027
FEV_1_/FVC	91.35 (86.45,96.55)	90.92 (85.35,97.15)	91.6 (87.95,92.95)	0.944
FEV_1_/FVC (% of predicted)	111/2 (106,116.5)	112 (105.75,117)	109.68 (107.65,113.02)	0.365
FVC<80% of predicted	53 (71.62%)	43 (75.44%)	17 (100%)	0.030
FEV_1_<80% of predicted	40 (54%)	27 (47.37%)	13 (76.47%)	0.066
FEV_1_/FVC <70% of predicted	0	0	0	
PEFR<80% of predicted	33 (44.6%)	25 (43.86%)	8 (47.06%)	0.815
FEF_25-75_<80% of predicted	12 (16.2%)	11 (19.3%)	1 (5.88%)	0.274
Spirometry interpretation				
Normal	13 (17.57%)	13 (22.8%)	0	0.031
Restrictive	61 (82.43%)	44 (77.2%)	17 (100%)	0.031
Obstructive	0	0	0	
Restrictive+Obstructive	0	0	0	
PaO_2_ (mmHg)	80.27 ± 8.51	81.53 ± 8.37	76.06 ± 7.8	0.019
PaCO_2_ (mmHg)	36.02 ± 2.55	35.97 ± 2.57	36.21 ± 2.53	0.738
ICU stay	10 (8,14.25)	9 (6.5,11)	21 (16,26)	<0.001
PCS	51.53 (41.9,54.92)	53.07 (47.41,55.5)	40.42 (36.87,44.58)	<0.001
MCS	57.38 (54.69,59.82)	57.42 (55.4,59.87)	56.12 (50.29,59.95)	0.365
6-MWTD	480.14 ± 85.7	515.75 ± 52.4	360.71 ± 64.94	<0.001
Percentage drop in saturation with 6-MWT	1 (1,1)	1 (1,1)	1 (1,2)	0.03

Out of 17 patients in Group B, abnormal FEV_1_, FVC, FEF_25-75_ and PEFR, and values were measured in 13 (76.47%), 17 (100%), 1 (5.88%) and 8 (47%) patients, respectively. None of the patients had abnormal FEV_1_/FVC values. On inference, all 17 patients had a restrictive pattern. Seven patients (41%) had mild restriction, 8 (47%) moderate and 2 (12%) moderately severe restriction upon on spirometry (Table 3 and Figure 3).

The median FEV_1_ (% of predicted), FVC (% of predicted) and FEF_25-75_ (% of predicted) were significantly different amongst Groups A and B (p=0.001, p<0.001 and p=0.027, respectively). There was no difference in FEV_1_/FVC (% of predicted) and PEFR (% of predicted) between the two groups (p=0.365 and p=0.639, respectively) (Table 3 and Figure 2). The PaO_2_ values (mean ± SD) were significantly higher in the Group A (81.53 ± 8.37 mmHg vs. 76.06 ± 7.8 mmHg in the Group B, p=0.019), whereas there was no difference in PaCO_2_ values between Groups A and B (35.97 ± 2.57 vs. 36.21 ± 2.53 mmHg; p=0.738) (Table 3). 

Secondary outcomes

6-MWT

The 6-MWT distance (mean ± SD) covered by participants in Group A and Group B was 515.75 ± 52.4 meters and 360.71 ± 64.94 meters, respectively (p<0.001). During the 6-MWT, the percentage drop in SpO_2_ (median [IQR]) was 1 (1,1) % in Group A and 1 (1,2) % in Group B (p=0.03), suggesting that the latter group had a greater fall in SpO_2_ after conducting the 6-MWT (Table 2, 3). None of the patients in either group had a drop in SpO_2_ more than 3% from the baseline.

Health-Related Quality of Life (HRQOL)

The PCS (median [IQR]) was 53.07 (47.41,55.5) in Group A and 40.42 (36.87,44.58) in the Group B (p<0.001). The MCS (median [IQR]) was 57.42 (55.4,59.87) in Group A and 56.12 (50.29,59.95) in Group B, (p=0.365) (Table 2, 3).

Forty-three patients in Group A and all patients in Group B were discharged on oral steroids. Oral anticoagulants were advised for 23 patients in Group A and all patients in Group B at discharge from the ICU. Seven patients in Group A and 1 patient in Group B required readmission for medical care during the first 3 months post-ICU discharge.

## Discussion

In the aftermath of the COVID-19 pandemic peaks, the burden of the sequelae in survivors is a new issue at hand. The probable pathophysiology associated with COVID-19 is a microvascular injury leading to alveolar damage and resulting in the loss of alveolar space. These changes may cause long-term pulmonary dysfunction [15]. From the understanding of other coronavirus-associated pneumonia, it has been observed that impaired lung function continues to afflict even patients who were not mechanically ventilated [16]. However, literature regarding pulmonary function and HRQOL post-ICU discharge in patients receiving mechanical ventilation is scarce.

To the best of our knowledge, our study is the first to have studied the consequences of invasive versus non-invasive oxygen therapy in COVID-19 survivors. In our study, we found that the majority of patients (77%) receiving non-invasive oxygen therapy (Group A), and all the patients (100%) receiving invasive mechanical ventilation (Group B) had a restrictive pattern on spirometry. Previous studies have reported the incidence of restrictive pattern in PFT in 21% to 27% of the patients, but their analysis included few or have excluded data of patients receiving NIV or invasive MV [9,17]. Huang et al, in their follow-up study 30-days after discharge, found that 12.3% of patients had a restrictive pattern in PFT [18]. However, 29.8% of patients included had severe or critical symptoms of supplemental oxygen (via any mode of oxygenation) whereas in our cohort only the patients who required oxygen (HFNC/NIV or MV) were recruited. This may be an indication of the presence of greater severity of the disease with a higher degree of lung parenchymal involvement.

In our study, 61 (82%) patients had abnormal lung function at 3 months post-ICU discharge. Studies have cited reduced spirometry values between 10% to 25% at 3 months after hospital discharge [19,20]. However, only 12% to 43% of their study population were admitted to the ICU. Similarly, Zhao et al. found lung abnormalities in 25% of patients but have excluded critical cases [21]. Our inclusion criteria of P/F ratio ≤ 200 mmHg ensured recruitment of patients with severe to critical COVID-19 pneumonia. The presence of reduced lung function has also been reported in 75.4% of survivors at 1-month follow-up [18].

The most frequent abnormality in our study was reduced FVC (81.08%) followed by FEV_1_ (54.05%) and PEFR (44.5%). Low FVC in the range of 24-28% and low FEV_1_ was found in 25% of patients in studies that also included data from oxygen requiring patients [18,20].

When we compared the median values of spirometric parameters between the group, FEV_1_ (% of predicted), FVC (% of predicted), and FEF_25-75_ (% of predicted) were significantly different amongst those requiring MV and those who could be managed on HFNC/NIPPV. However, there was no difference in FEV_1_/FVC (% predicted) and PEFR (% of predicted) between the groups. Anastosio et al. reported a significant difference in FEV_1_%, FVC%, FEV1/FVC% and PEFR% of predicted values when compared between those requiring MV and those not requiring MV [22]. Lerum et al. reported no significant difference in FEV_1_, FVC, and FEV_1_/FVC ratio at 3 months between ICU (15 patients with 9 requiring MV) and non-ICU patients (88 patients) [23]. The ICU admission in this study was based on unsatisfactory SpO_2_ with a nasal cannula or a non-rebreather mask. In a study conducted by Mo et al., at the time of discharge from the hospital, there was no significant difference in the spirometry parameters amongst those with pneumonia and severe pneumonia survivors. They have also excluded the critical cases (those patients requiring ICU admission and MV) [24]. In mechanically ventilated patients, the decreased FVC may be explained by the combination of prolonged use of muscle relaxation, deep sedation, and lack of spontaneous respiratory movements for several days that might be detrimental to overall compliance of the respiratory muscles. Huang et al. also did not find any significant difference in FEV_1_, FVC, and FEV_1_/FVC ratio between severe and non-severe disease survivors at 30 days post discharge [18]. Follow-up studies of SARS-recovered patients highlight the presence of lung function impairment for months [25].

Spirometry is a practical tool for assessing lung function recovery post discharge. Inclusion of PFT as part of follow-up care of COVID-19 pneumonia is hence recommended [26].

The 6-MWT along with spirometry helps in evaluating the underlying impairment in the lung function that may hinder complete recovery after COVID-19 pneumonia. These simple tests might potentially aid in identifying the patients in greatest need of pulmonary rehabilitation facilities. The mean distance covered by patients in our study was 480 meters, which was less than that reported in previous studies [17, 22]. The shorter 6-MWT distance covered in our study may be because the patients were sicker with lower mean PEFR at admission. The 6-MWT correlates with the severity of respiratory parameters and capacity [27]. In addition, our study observed a significant difference in 6-MWT between the groups - 515.75 ± 52.4 meters in Group A vs. 360.71 ± 64.94 meters in Group B; p<0.001). Lerum et al. found a significant difference in distance covered between ICU and non-ICU survivors [23]. Another study reported a significant difference in 6-MWT which co-related with a decreased exercise tolerance to the severity of respiratory symptoms [18].

HRQOL may depict the effect of the residual disease on the individual’s perception of mental and physical well-being. In our study the MCS and PCS scores (median [IQR]) were 57.38 (54.69,54.92) and 51.53 (41.9,54.92) respectively. These scores were better than those reported previously [22]. Normal mental health was reported despite extreme uncertainty during the COVID-19 may be due to contentment for having survived the ordeal. On comparison between the groups, there were significant differences in the PCS score with no difference in the MCS. Physical debilitation in the MV group is expected owing to the prolonged use of muscle relaxants and sedation, which are predisposing factors to critical illness myopathy and takes time to recover [28].

There are certain limitations to our study. Firstly, the lack of baseline spirometry values prior to the onset of illness makes comparisons difficult. We cannot comment if these abnormalities were present prior to the onset of COVID-19 pneumonia. We could not include data from all the survivors due to loss to follow-up or even due to perception of complete recovery and hesitancy to return to the “COVID” hospital. Our study included patients who were sicker as compared to other studies. The physical weakness may also have contributed to the shorter 6-MWT distance. Also, the SF-12 questionnaire used in this study does not encompass all aspects of mental health. Most studies have included or classified patients as severe and non-severe or compared characteristics of non-pneumonia and those with pneumonia. Our study is novel in including only the patients with severe or critical COVID-19, requiring non-invasive oxygen therapy or invasive mechanical ventilation and comparing spirometry values, physical and mental effects 3 months post-discharge from the ICU. 

## Conclusions

Our study shows that patients requiring mechanical ventilation for the management of COVID-19 pneumonia are at greater risk of pulmonary function abnormalities and physical limitation after discharge from the hospital. Larger follow-up studies or multicentre data on this subset of patients will further support our findings.

A strict follow-up protocol is essential after recovery from mechanical ventilation for COVID-19 pneumonia as the incidence of functional limitations is high. There may be a greater need for rehabilitative therapies for such patients.

## References

[1] Grasselli G, Zangrillo A, Zanella A (2020). Baseline characteristics and outcomes of 1591 patients infected with SARS-CoV-2 admitted to ICUs of the Lombardy Region, Italy. JAMA.

[2] Vitacca M, Clini E, Rubini F, Nava S, Foglio K, Ambrosino N (1996). Non-invasive mechanical ventilation in severe chronic obstructive lung disease and acute respiratory failure: short- and long-term prognosis. Intensive Care Med.

[3] Storgaard LH, Hockey HU, Laursen BS, Weinreich UM (2018). Long-term effects of oxygen-enriched high-flow nasal cannula treatment in COPD patients with chronic hypoxemic respiratory failure. Int J Chron Obstruct Pulmon Dis.

[4] Antonelli M, Conti G, Proietti R (10.1007/978-3-642-59467-0_44). Non-invasive ventilation in acute hypoxemic respiratory failure. Yearbook of Intensive Care and Emergency Medicine 2001.

[5] Wunsch H (2020). Mechanical ventilation in COVID-19: interpreting the current epidemiology. Am J Respir Crit Care Med.

[6] Nalbandian A, Sehgal K, Gupta A (2021). Post-acute COVID-19 syndrome. Nat Med.

[7] Hopkins RO, Weaver LK, Collingridge D, Parkinson RB, Chan KJ, Orme JF Jr (2005). Two-year cognitive, emotional, and quality-of-life outcomes in acute respiratory distress syndrome. Am J Respir Crit Care Med.

[8] Fan E, Dowdy DW, Colantuoni E (2014). Physical complications in acute lung injury survivors: a two-year longitudinal prospective study. Crit Care Med.

[9] van der Sar-van der Brugge S, Talman S, Boonman-de Winter L, de Mol M, Hoefman E, van Etten RW, De Backer IC (2021). Pulmonary function and health-related quality of life after COVID-19 pneumonia. Respir Med.

[10] Pellegrino R, Viegi G, Brusasco V (2005). Interpretative strategies for lung function tests. Eur Respir J.

[11] (2002). ATS statement: guidelines for the six-minute walk test. Am J Respir Crit Care Med.

[12] Wilkerson RG, Adler JD, Shah NG, Brown R (2020). Silent hypoxia: a harbinger of clinical deterioration in patients with COVID-19. Am J Emerg Med.

[13] Ware J Jr, Kosinski M, Keller SD (1996). A 12-item short-form health survey: construction of scales and preliminary tests of reliability and validity. Med Care.

[14] Gandek B, Ware JE, Aaronson NK (1998). Cross-validation of item selection and scoring for the SF-12 Health Survey in nine countries: results from the IQOLA Project. International Quality of Life Assessment. J Clin Epidemiol.

[15] Laveneziana P, Straus C, Meiners S (2021). How and to what extent immunological responses to SARS-CoV-2 shape pulmonary function in COVID-19 patients. Front Physiol.

[16] Ahmed H, Patel K, Greenwood DC (2020). Long-term clinical outcomes in survivors of severe acute respiratory syndrome and Middle East respiratory syndrome coronavirus outbreaks after hospitalisation or ICU admission: A systematic review and meta-analysis. J Rehabil Med.

[17] Truffaut L, Demey L, Bruyneel AV, Roman A, Alard S, De Vos N, Bruyneel M (2021). Post-discharge critical COVID-19 lung function related to severity of radiologic lung involvement at admission. Respir Res.

[18] Huang Y, Tan C, Wu J (2020). Impact of coronavirus disease 2019 on pulmonary function in early convalescence phase. Respir Res.

[19] Méndez R, Latorre A, González-Jiménez P (2021). Reduced diffusion capacity in COVID-19 survivors. Ann Am Thorac Soc.

[20] Sibila O, Albacar N, Perea L (2021). Lung function sequelae in COVID-19 patients 3 months after hospital discharge. Arch Bronconeumol.

[21] Zhao YM, Shang YM, Song WB (2020). Follow-up study of the pulmonary function and related physiological characteristics of COVID-19 survivors three months after recovery. EClinicalMedicine.

[22] Anastasio F, Barbuto S, Scarnecchia E (2021). Medium-term impact of COVID-19 on pulmonary function, functional capacity and quality of life. Eur Respir J.

[23] Lerum TV, Aaløkken TM, Brønstad E (2021). Dyspnoea, lung function and CT findings 3 months after hospital admission for COVID-19. Eur Respir J.

[24] Mo X, Jian W, Su Z (2020). Abnormal pulmonary function in COVID-19 patients at time of hospital discharge. Eur Respir J.

[25] Hui DS, Joynt GM, Wong KT (2005). Impact of severe acute respiratory syndrome (SARS) on pulmonary function, functional capacity and quality of life in a cohort of survivors. Thorax.

[26] George PM, Barratt SL, Condliffe R (2020). Respiratory follow-up of patients with COVID-19 pneumonia. Thorax.

[27] Singh SJ, Puhan MA, Andrianopoulos V (2014). An official systematic review of the European Respiratory Society/American Thoracic Society: measurement properties of field walking tests in chronic respiratory disease. Eur Respir J.

[28] Zhou C, Wu L, Ni F, Ji W, Wu J, Zhang H (2014). Critical illness polyneuropathy and myopathy: a systematic review. Neural Regen Res.

